# *MYD88*-Mutated Chronic Lymphocytic Leukaemia/Small Lymphocytic Lymphoma as a Distinctive Molecular Subgroup Is Associated with Atypical Immunophenotypes in Chinese Patients

**DOI:** 10.3390/jcm12072667

**Published:** 2023-04-03

**Authors:** Yafei Mu, Xijie Fan, Tao Chen, Yuhuan Meng, Junwei Lin, Jiecheng Yuan, Shihui Yu, Yuxin Chen, Lingling Liu

**Affiliations:** 1 Guangzhou KingMed Transformative Medicine Institute Co., Ltd., Guangzhou 510320, China; 2 KingMed School of Laboratory Medicine, Guangzhou Medical University, Guangzhou 510180, China; 3 Guangzhou KingMed Diagnostics Group Co., Ltd., Guangzhou 510320, China; 4 Clinical Genome Center, Guangzhou KingMed Center for Clinical Laboratory Co., Ltd., Guangzhou 510320, China; 5 Department of Hematology, The Third Affiliated Hospital of Sun Yat-sen University and Sun Yat-sen Institute of Hematology, Guangzhou 510630, China

**Keywords:** chronic lymphocytic leukaemia/small lymphocytic lymphoma (CLL/SLL), *MYD88* variants, genetic aberrations, immunophenotypes, mantle cell lymphoma (MCL)

## Abstract

Chronic lymphocytic leukaemia/small lymphocytic lymphoma (CLL/SLL) is a heterogeneous disease in Western and Chinese populations, and it is still not well characterized in Chinese patients. Based on a large cohort of newly diagnosed CLL/SLL patients from China, we investigated immunophenotypes, genetic abnormalities, and their correlations. Eighty-four percent of the CLL/SLL patients showed typical immunophenotypes with scores of 4 or 5 points in the Royal Marsden Hospital (RMH) scoring system (classic group), and the remaining 16% of patients were atypical with scores lower than 4 points (atypical group). Trisomy 12 and variants of *TP53*, *NOTCH1*, *SF3B1*, *ATM*, and *MYD88* were the most recurrent genetic aberrations. Additionally, unsupervised genomic analysis based on molecular genetics revealed distinctive characteristics of *MYD88* variants in CLL/SLL. By overlapping different correlation grouping analysis from genetics to immunophenotypes, the results showed *MYD88* variants to be highly related to atypical CLL/SLL immunophenotypes. Furthermore, compared with mantle cell lymphoma (MCL), the genetic landscape showed potential value in clinical differential diagnosis of atypical CLL/SLL and MCL patients. These results reveal immunophenotypic and genetic features, and may provide insights into the tumorigenesis and clinical management of Chinese CLL/SLL patients.

## 1. Introduction

Chronic lymphocytic leukaemia/small lymphocytic lymphoma (CLL/SLL) is one of the most common leukaemias in the Western world [1,2,3,4]. Over the past decades, there has been increasing appreciation of the impact of genetic abnormalities on the onset and progression of this disease from studies in Western populations [1,2,3,4]. CLL/SLL is a hematologic neoplasm that is heterogeneous in populations from different backgrounds in the West and Asia, particularly in individuals from China [5,6,7,8,9]. Notably, several apparent differences between Chinese CLL/SLL patients and those from Western countries have been reported, such as age distribution, chromosomal aberrations, gene variants, IGHV family usage, and treatment outcomes [5,6,7,8,9,10,11,12], and additional findings need further investigation. While studies including large cohorts of Chinese patients or population-based studies in mainland China with CLL/SLL are rare, an understanding of the features of CLL/SLL in Chinese patients will help guide future research on disease aetiology, pathogenesis, biology, and advanced clinical application.

Myeloid differentiation primary response protein 88 (MYD88) is a general adaptor protein, and *MYD88* variants always drive the constitutive activation of nuclear factor-κB (NF-κB), which commonly occurs in various mature B-cell neoplasms (MBNs) [13,14]. High incidence of *MYD88* variants has been detected in lymphoplasmacytic lymphoma/Waldenstrom macroglobulinemia (LPL/WM) (~95%), and it is also occasionally reported in diffuse large B-cell lymphoma (DLBCL) (20~30%), CLL/SLL (3~10%), and marginal zone B-cell lymphoma (MZBL) (5~7%), but rarely reported in other subtypes [13,14,15]. Clinically, the presence of the *MYD88* L265P variant as an independent criterion can render the diagnosis of LPL/WM either more or less likely, and it is less frequently used as a diagnostic or prognostic marker in other subtypes.

In this study, we retrospectively evaluated 761 newly diagnosed CLL/SLL patients in our institution. Based on the Chinese population’s genetic background, we aimed to reveal the immunophenotype and genetic clinical features of CLL/SLL patients and provide new insights into clinical management and the development of novel therapeutic strategies for this disease.

## 2. Materials and Methods

### 2.1. Patients and Sample Collection

From 1 January 2018 to 31 December 2021, a total of 761 newly diagnosed CLL/SLL patients and 74 newly diagnosed mantle cell lymphoma (MCL) patients were retrospectively analysed in our laboratory. Each patient was diagnosed and classified based on the 2016 revision of the World Health Organization classification of lymphoid neoplasms. The CLL/SLL Royal Marsden Hospital (RMH) score is a classic immunological diagnostic system that is widely used in clinical practice. If the RMH score is ≥4, the diagnosis of CLL/SLL is supported, and the case is considered typical CLL/SLL. The RMH scoring system was used for CLL/SLL grouping in this study. CLL/SLL patients with scores of 4 or 5 points were considered to have classic CLL/SLL immunophenotypes and were grouped into the classic group (C-CLL/SLL); CLL/SLL patients with scores lower than 4 points were considered to have atypical CLL/SLL immunophenotypes and were grouped into the atypical group (A-CLL/SLL).

### 2.2. Flow Cytometry (FC)

Flow cytometry (FC) was performed on fresh bone marrow aspiration or peripheral blood samples. FC panels were designed and utilized according to the referring doctors’ orders, and among the available markers, we selected cell surface clusters of differentiation (CD) markers associated with CLL/SLL, including CD5, CD10, CD19, CD20, CD22, CD23, CD79b, CD200, FMC7, sIg-Kappa, and sIg-Lambda, for further analysis in this study. Five-colour analysis used FITC (fluorescein-isothiocyanate)-, PE (phycoerythrin)-, ECD-, PC5 (PE-Cy5 conjugates)-, and PC7 (PE-Cy7 conjugates)-labelled mouse anti-human fluorescent monoclonal antibodies, and cell surface staining was performed according to conventional protocols. The expression level of each CD marker was rated “negative”, “dim”, or “bright”. “Positive” included “dim” and “bright”. FC was performed on a Cytomics FC500 Cytometer (Beckman Coulter, Brea, CA, USA), and data were analysed with FCS express flow cytometry software (De Novo Software, Los Angeles, CA, USA).

### 2.3. Next-Generation Sequencing (NGS) and Variant Curation

An NGS panel consisting of 175 genes associated with hematological malignancy (called the 175-Panel) (Supplementary Table S1) was used for the analysis of all patient samples in this study. DNA was extracted from formalin-fixed paraffin-embedded samples of tissue or from bone marrow samples and peripheral blood samples using a QIAamp DNA Mini Kit (Qiagen, Hilden, Germany). A hybrid capture method for library preparation was adopted, using IDT probes (Integrated DNA Technologies, Coralville, IA, USA) and a KAPA Library Amplification Kit (Kapa Biosystems, Wilmington, MA, USA). DNA sequencing was performed on an Illumina NovaSeq6000 system (Illumina, San Diego, CA, USA) according to the manufacturer’s recommendations. Variant detection was performed using the Somatic Variant Caller Algorithm from Illumina with default filtering settings. Variant curation was performed according to the Standards and Guidelines for the Interpretation and Reporting of Sequence Variants in Cancer. Variants with strong clinical significance (Tier I) and variants with potential clinical significance (Tier II) were the focus of this study.

### 2.4. Immunoglobulin Heavy Chain (IGH) Variable (V) Region Mutational Status Analysis

Two methods, Sanger sequencing (before Mar. 2019) and NGS (Mar. 2019 to present), were used for IGHV mutational status analysis. DNA was extracted from formalin-fixed paraffin-embedded samples of tissue or from bone marrow samples and peripheral blood samples following conventional protocols. Capillary electrophoresis was used for IGH clonality analysis. IGHV status analysis was performed by following the IGH Somatic Hypermutation Assay v2.0 protocol (Invivoscribe, San Diego, CA, USA). Sequence data were analysed using GENEMAPPER software and compared to reference sequences in the NCBI database. An IGHV Leader Somatic Hypermutation Assay (Invivoscribe, San Diego, CA, USA) was used to identify and track clonal B-cell IGH rearrangements using NGS with the Illumina MiSeq platform and to assess the extent of somatic hypermutation in the variable heavy chain gene sequence in the samples. In addition, 98% IGHV gene identity was the cut-off point for IGHV mutational status.

### 2.5. Chromosomal Karyotype and Fluorescence In Situ Hybridization (FISH) Analysis

Conventional G-banded chromosomal analysis was performed on unstimulated bone marrow aspiration or peripheral blood cultured for 47 h or 71 h using standard techniques. No fewer than 20 meta-phases were karyotyped. An abnormal karyotype (AK) was defined by the presence of at least one chromosomal abnormality, and a complex karyotype (CK) was defined by the presence of three or more chromosomal abnormalities in a single clone. Vysis probes (Abbott Molecular, Des Plaines, IL, USA) for CEP12, 11q22.3, 17p13.1, and 13q14.2 were used for FISH analysis. Hybridization and post-hybridization washes were performed by following the corresponding manufacturer’s protocol based on the ThermoBrite system (Leica Biosystems, Buffalo Grove, IL, USA), and the cells were counterstained with DAPI II (marrow aspiration and peripheral blood) or DAPI I (formalin-fixed paraffin-embedded tissue). The results were reported according to the International System for Human Cytogenetic Nomenclature (ISCN) 2016.

### 2.6. Statistical Analysis

Statistical analysis was performed by using R (R version 4.1.0). Variant analysis was performed using the R software package “maftools”. Unsupervised analysis was performed to cluster CLL/SLL patients into genomic subgroups by hierarchical clustering using Ward’s criteria based on the silhouette value. Correlation analysis was performed using the R software package “psych”. The χ^2^ or Fisher’s exact test was used for the between-group comparison of discrete variables. A two-sample t-test was used for the between-group comparison of the means. *p* values < 0.05 were considered statistically significant.

## 3. Results

### 3.1. A High Proportion of Chinese CLL/SLL Patients Had Atypical Immunophenotypes

The RMH scoring system plays a key role in assisting in the diagnosis of CLL/SLL patients; therefore, we investigated its clinical application in our Chinese CLL/SLL cohort. In 761 newly diagnosed CLL/SLL patients (the male-to-female ratio was 1.92:1, and the average age at diagnosis was 64.8), the RMH scores of 618 patients were assessable. A total of 519 (84.0%) of these CLL/SLL patients received scores of 4 or 5 points based on the CLL/SLL RMH scoring system (classic group), and 99 (16.0%) CLL/SLL patients received scores lower than 4 points (atypical group) (Table 1). CLL/SLL patients with atypical immunophenotypes represented a high percentage of the Chinese cohort.

### 3.2. Mutation Landscape of Chinese CLL/SLL Patients

NGS has gradually become a routine clinical tool for the analysis of CLL/SLL patients. Here, we summarized the mutation spectrum and investigated potential molecular subtypes based on a large Chinese CLL/SLL cohort. Samples from all 761 patients were analysed using NGS, and a total of 1368 variants were found in 564 (74.1%) CLL/SLL patients. *TP53* (13.5%), *NOTCH1* (12.0%), *SF3B1* (10.0%), *ATM* (9.3%), and *MYD88* (8.5%) were the most recurrently mutated genes in the entire CLL/SLL cohort.

**Figure 1:**
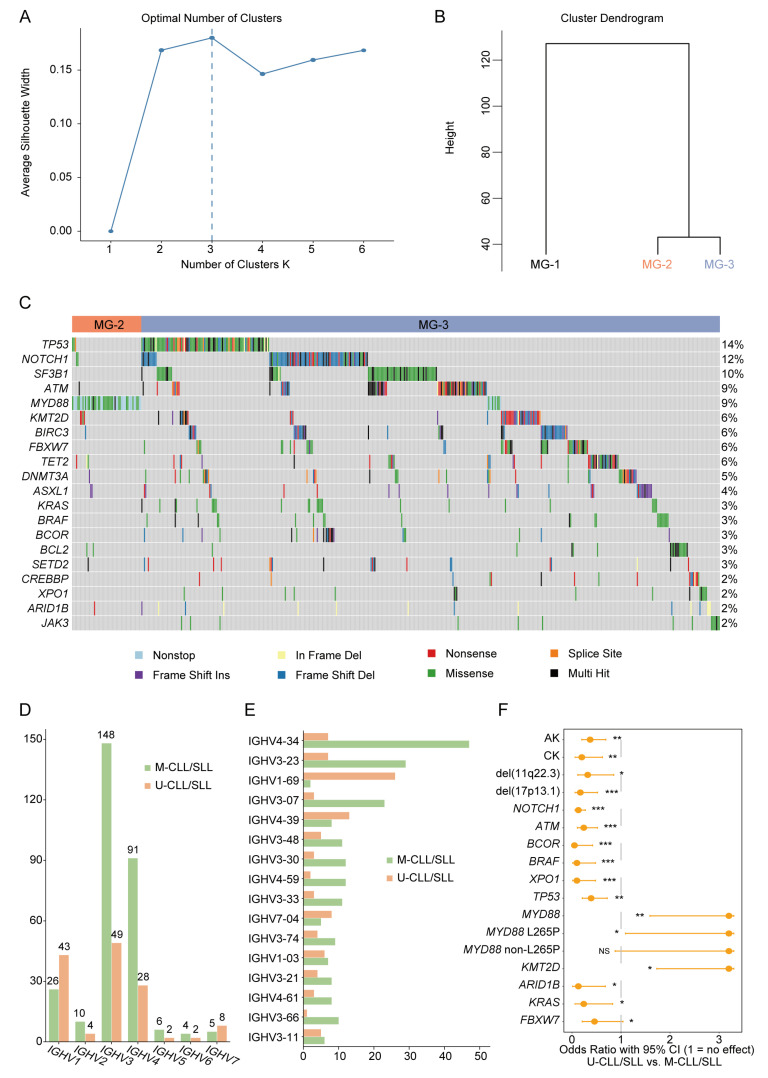
Genetic abnormalities and IGHV family and subfamily usage in CLL/SLL. (**A**) Optimal number of clusters based on the highest silhouette value. (**B**) Three novel molecular subgroups unveiled through unsupervised genomic analysis. (**C**) Variant landscape of 761 newly diagnosed CLL/SLL patients. (**D**) IGHV family usage and distribution. (**E**) IGHV subfamily usage and distribution. (**F**) Genetic abnormality differences between IGHV M-CLL/SLL and IGHV U-CLL/SLL. M-CLL/SLL: hypermutated IGHV CLL/SLL; U-CLL/SLL: non-hypermutated IGHV CLL/SLL; AK: abnormal karyotype; CK: complex karyotype; ***: *p* value < 0.001; **: *p* value < 0.01; *: *p* value < 0.05; NS: no significance.

Based on the 175-Panel variant screen, we performed unsupervised genomic analysis, unveiling three novel molecular CLL/SLL subgroups as determined by the silhouette value (Figure 1A,B). MG-1 included CLL/SLL patients with no variants. MG-2 was characterized by the full presence of *MYD88* variants, and the remaining case was MG-3 (Figure 1C). In this Chinese CLL/SLL cohort, CLL/SLL patients with *MYD88* variants showed a distinctive molecular grouping status in the Chinese CLL/SLL cohort.

### 3.3. MYD88 Variants Related to Hypermutated IGHV Status

IGHV family usage and somatic hypermutation are important prognostic factors in CLL/SLL. IGH clonality and somatic hypermutation status were analysed in 483 patients. The frequency of IGHV gene family usage was determined for 460 (95.2%) patients, while 50 (10.9%) of these patients were nonassessable owing to multiple clonotypes. A total of 430 sequences were obtained in the remaining 410 (89.1%) patients, as 20 (4.9%) patients held two clones (Figure 1D,E). A hypermutated IGHV status (IGHV M-CLL/SLL) in 279 (68.0%) patients and a non-hypermutated status (IGHV U-CLL/SLL) in 129 (31.5%) patients were detected. Two (0.5%) patients were nonassessable owing to the conflicting IGHV status of two clones. A total of 413 variants were found in 279 patients in the IGHV M-CLL/SLL group, and 328 variants were found in 129 patients in the IGHV U-CLL/SLL group.

Then, we investigated the potential impact of *MYD88* variants on patient prognosis based on the above findings. *MYD88* variants (*p* = 0.002) were predominant in patients of IGHV M-CLL/SLL, indicating a good prognosis for CLL/SLL patients (Figure 1F). Interestingly, when we reviewed relevant studies from the past few decades in China, we found that the frequency of IGHV1-69 gene usage was high in our CLL/SLL cohort and rose yearly in Chinese CLL/SLL patients (Table 2).

### 3.4. MYD88 Variants Related to Abnormal Karyotypes

Cytogenetic analysis of CLL/SLL patients is necessary for clinical management. Therefore, we summarized the cytogenetic abnormalities in Chinese CLL/SLL patients. Conventional chromosomal G banding analysis was performed for 497 patients. Abnormal karyotypes (AK) were found in 87 (17.5%) patients, and complex karyotypes (CK) were observed in 22 (4.4%) patients. Trisomy 12 (6.6%) was the most frequent abnormality. Among the FISH group, trisomy 12 (20.5%), del(11q22.3) (11.1%), del(17p13.1) (8.1%), and del(13q14.2) (23.0%) were confirmed.

**Figure 2:**
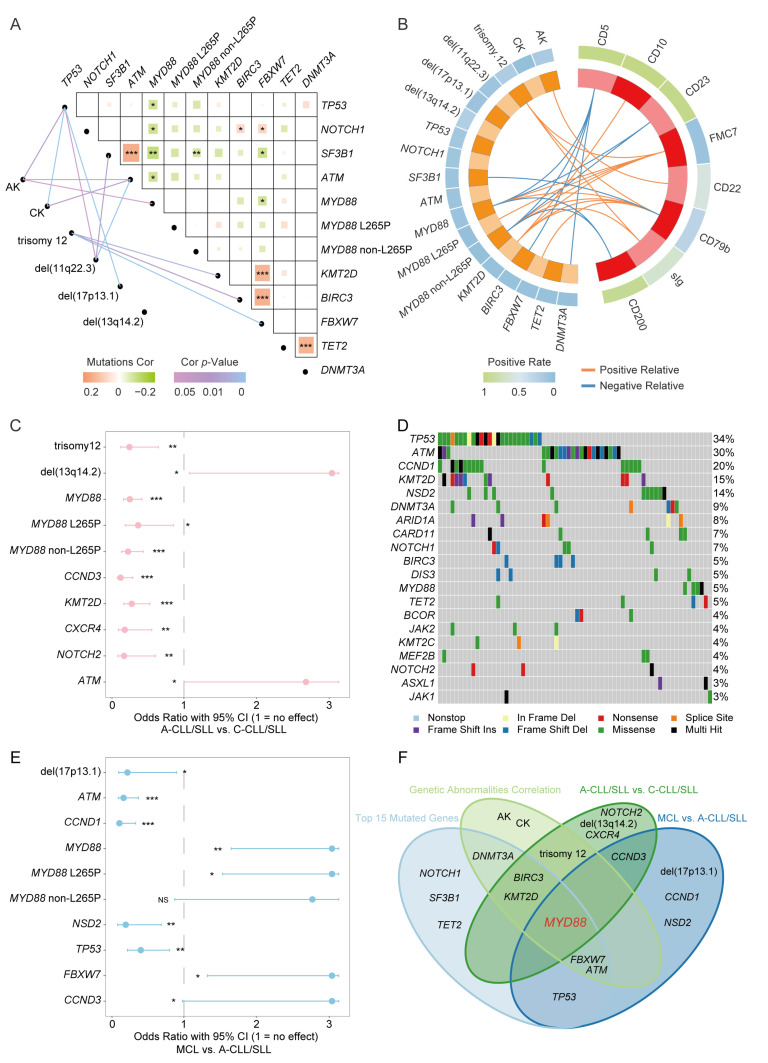
Correlation analysis between genetic abnormalities and immunophenotypes in CLL/SLL. (**A**) Gene variant somatic interaction and correlation with cytogenetic aberrations. (**B**) Genetic abnormalities associated with CD markers. (**C**) Genetic abnormality differences between atypical CLL/SLL and classic CLL/SLL. (**D**) Mutation landscape in 74 newly diagnosed MCL patients. (**E**) Gene variant differences between MCL and atypical CLL/SLL. (**F**) Genetic abnormality overlapping between the mentioned grouping analysis. A-CLL/SLL: atypical immunophenotype CLL/SLL; C-CLL/SLL: classic immunophenotype CLL/SLL; ***: *p* value < 0.001; **: *p* value < 0.01; *: *p* value < 0.05; NS: no significance.

Based on the distinctive status of *MYD88* variants mentioned above, we analysed their correlations with cytogenetic aberrations. AK (*p* = 0.006) and del(17p13.1) (*p* < 0.001) showed significant differences between the three novel molecular subgroups, and AK (*p* = 0.048) showed a significant correlation with *MYD88* variants (Table 3, Figure 2A). In addition, some other variants were associated with certain cytogenetic abnormalities. AK was associated with *ATM* and *TP53* variants, CK was associated with *ATM* and *TP53* variants, trisomy 12 was associated with *BIRC3*, *FBXW7*, and *KMT2D* variants, del(11q22.3) was associated with *ATM*, *SF3B1*, and *TP53* variants, and del(17p13.1) was associated with *TP53* variants (Figure 2A).

### 3.5. MYD88 Variants Related to Atypical CLL/SLL Immunophenotypes

Next, we analysed the correlations between *MYD88* variants and immunophenotypes. The expression of seven immunophenotypic markers (CD5, CD23, FMC7, CD22, CD79b, and sIg) in the RMH scoring system and two CLL/SLL identification-related markers (CD10 and CD200) were used for correlation analysis. First, CD23 (*p* = 0.03), FMC7 (*p* < 0.001), CD79b (*p* = 0.007), and CD200 (*p* < 0.001) showed significant differences between the three novel molecular subgroups (Table 3). CD5 negativity (*p* = 0.04), CD23 negativity (*p* = 0.03), FMC7 positivity (*p* < 0.001), high CD79b expression (*p* < 0.01), and CD200 negativity (*p* < 0.001) showed significant correlations with *MYD88* variants (Figure 2B).

Furthermore, we grouped the CLL/SLL patients into a classic group and an atypical group according to the CLL/SLL RMH scoring system, and we found that *MYD88* (*p* < 0.001) was predominant in the atypical group of patients (Figure 2C). Above all, *MYD88* variants showed a strong correlation with atypical CLL/SLL immunophenotypes.

### 3.6. MYD88 Variants May Be a Potential Differential Diagnostic Marker between Atypical CLL/SLL and Mantle Cell Lymphoma (MCL)

Clinically, patients of CLL/SLL always need to be differentially diagnosed with mantle cell lymphoma (MCL), and it is especially important to distinguish CLL/SLL with RMH scores lower than 4 points (atypical CLL/SLL) from MCL. Based on the significant correlation between *MYD88* variants and atypical CLL/SLL, we further explored its clinical application potential in the differential diagnosis between atypical CLL/SLL and MCL. In total, 74 MCL patients were assessed. *TP53* (33.8%), *ATM* (30.0%), *CCND1* (20.3%), *KMT2D* (14.9%), and *NSD2* (13.5%) were the most recurrently mutated genes in the MCL cohort (Figure 2D). Compared with MCL, *MYD88* variants (*p* = 0.001) were predominant in patients of atypical CLL/SLL (Figure 2E). By overlapping different correlation grouping analysis from genetics to immunophenotypes, *MYD88* variants showed significant and distinctive characteristics in CLL/SLL, and may be a potential differential diagnosis marker for MCL (Figure 2F).

## 4. Discussion

In this study, we unveiled the clinical characteristics of Chinese CLL/SLL patients, and based on genetic and immunophenotype analysis, we revealed some novel findings. The median age of our CLL/SLL cohort was similar to that of patients from other centers in China, and the patients were younger than CLL/SLL patients from the West [7,8,9]. To determine the immunophenotypes of Chinese CLL/SLL patients, we used the RMH scoring system. Eighty-four percent of CLL/SLL patients scored 4 or 5 points (classic group), while 16% scored lower than 4 points (atypical group), which was similar to the results from previous Chinese and other Asian studies but different from studies in Western populations, in which only approximately 5% of CLL/SLL patients from the West scored lower than 4 points [19,20,21,22]. This finding indicated population bias in the usability of the RMH scoring system to diagnose CLL/SLL in Chinese patients. On the other hand, it also revealed a high proportion of atypical CLL/SLL patients in the Chinese CLL/SLL cohort, which may be associated with the genetic differences between Chinese and Western CLL/SLL patients.

Based on analysis of molecular genetics, *TP53*, *NOTCH1*, *SF3B1*, *ATM*, and *MYD88* were the most recurrently mutated genes in the entire CLL/SLL cohort, and three novel molecular subgroups were identified through unsupervised genomic analysis. *MYD88* variants were associated with a distinctive grouping status in the CLL/SLL cohort. The incidence of *MYD88* variants has been discordant according to previous research, with variant frequencies ranging from 3 to 20% in different studies [15,17,18]. In our cohort, *MYD88* variants showed significant differences in the incidence of immunophenotype grouping. The high incidence of *MYD88* variants in the atypical CLL/SLL patients indicated that in addition to population background differences, immunophenotype grouping bias may also be one of the reasons for the discordant incidence of *MYD88* variants in different studies. Furthermore, *MYD88* variants predominated in the IGHV M-CLL/SLL patients, indicating a potentially favourable prognosis in *MYD88*-mutated CLL/SLL patients, but previous studies showed that the prognostic impact of *MYD88* variants in CLL/SLL was still discordant—multiple studies in CLL/SLL have shown *MYD88* variants to have a favourable prognosis or no association with the course of disease, whereas others have shown *MYD88* variants to be associated with a poor prognosis [23,24,25]. Notably, atypical CLL/SLL was shown to be associated with an inferior outcome in previous studies, combined with the significant correlation between *MYD88* variants and atypical immunophenotypes in this study, suggesting the potential influence of immunophenotype grouping bias on the prognostic impact of *MYD88* variants [19,26]. Our novel finding on *MYD88* variants provides a new perspective on its discordance both in mutation frequency and prognosis. *KMT2D* variants are also a different type of molecular abnormality that has rarely been reported in CLL/SLL patients from the West, but is highly recurrent in Chinese CLL/SLL patients [12,27]. This study further confirmed the high recurrence of *KMT2D* variants in Chinese CLL/SLL patients, indicating that *KMT2D* variants are probably unique molecular characteristics in this subset of Chinese CLL/SLL patients. The tumourigenesis characteristics associated with *KMT2D* variants and their impact on clinical prognosis need more investigation.

IGHV1-69 is a significant geographical distribution-related V gene segment recurrently selected in the West (~15%) but rare in Asian and Chinese CLL/SLL patients (1~10%) [5,18,28]. Due to the fact that the presence of IGHV1-69 is associated with non-hypermutated IGHV status, IGHV1-69 is related to unfavourable prognosis. However, unlike IGHV3-21, which is identified as an independent predictor for prognosis in CLL/SLL, IGHV1-69 gene usage per se does not seem to be predictive of progressive disease, and progression is primarily related to the IGHV status [29,30]. In addition, IGHV1-69 has been reported to be closely linked to certain viruses and viral infections [31]. The increasing trend of IGHV1-69 gene usage in Chinese CLL/SLL patients may not affect its clinical prognostic assessment, but suggests that certain geographical, environmental, or biological factors may have an enhanced and lasting impact on its tumourigenesis and biological process.

Through correlation analysis, we further revealed the interaction between genetics and the expression of each CD marker in Chinese CLL/SLL patients and then compared classic and atypical CLL/SLL based on the immunophenotypes. These findings reveal the characteristics of Chinese CLL/SLL patients and are valuable for tumorigenesis research, functional studies, and clinical applications.

The clinical RMH scoring system is routinely applied for the differential diagnosis of patients with CLL/SLL and MCL [2,4]. Nevertheless, the high proportion of atypical CLL/SLL patients has limited the clinical application of this scoring system in Chinese patients. Therefore, based on the distinctive status of *MYD88* variants, we provide a potential new method of molecular genetic analysis to address the difficulties of using the RMH scoring system for the differential diagnosis of atypical CLL/SLL and MCL. This approach will be valuable in assisting in the routine clinical differential diagnosis of CLL/SLL patients.

Our study has multiple limitations that should be carefully considered. First, due to differences in individual clinical conditions, not all patients in this study underwent a comprehensive test at the time of their visit. In addition, the methodology of the IGHV mutational status analysis was updated after laboratory verification during the study. These restrictions may have certain impacts on test consistency and further statistical analysis. Last, the different sample types from patients in immunophenotyping may also have some impacts on the statistical analysis due to biology and cellular microenvironment differences. Nevertheless, based on such a large sample cohort, this study still provided reliable results, which are valuable for both functional studies and clinical applications.

In conclusion, Chinese CLL/SLL patients showed genetic and immunophenotypic features that were different from those of CLL/SLL patients in Western countries. The high proportion of atypical CLL/SLL patients, high incidence of *MYD88* and *KMT2D* variants, and significant correlation between *MYD88* variants and atypical immunophenotypes suggested that in addition to classic diagnostic and prognostic assessment systems, more comprehensive population-related characteristics should be taken into consideration in the clinical diagnosis, management, and treatment strategies of CLL/SLL. It is clear that several distinctive differences exist in Chinese CLL/SLL patients, but we still believe that the overall characteristics of CLL/SLL patients are consistent in China, the rest of Asia, and Western countries. The findings of this study may provide new insights to explain the discordance present in the West, and the key will be how to use these differences for the clinical diagnosis and treatment of Chinese patients. Some issues and challenges require further research in the future.

## Figures and Tables

**Table 1:** Summary of the patients’ general clinical characteristics and immunophenotypes.

Patient Characteristics	CLL/SLL
Total patients, n	761
Age, median (range)	64.8 (20–92)
Sex, n	
Female	261 (of 761, 34.3%)
Male	500 (of 761, 65.7%)
RMH, n	
RMH ≥ 4 points (classic group)	519 (of 618, 84.0%)
RMH < 4 points (atypical group)	99 (of 618, 16.0%)
CD, n	
CD5 (pos)	724 (of 726, 99.7%)
CD10 (neg)	724 (of 725, 99.9%)
CD23 (pos)	709 (of 720, 98.5%)
FMC7 (neg)	658 (of 720, 91.4%)
CD22 (neg/dim)	145 (of 360, 40.3%)
CD79b (neg/dim)	427 (of 600, 71.2%)
sIg (neg/dim)	241 (of 724, 33.3%)
CD200 (pos)	558 (of 587, 95.1%)

**Table 2:** IGHV subfamily usage in recent Chinese CLL/SLL studies.

	2008 (n = 65) [10]		2009 (n = 46) [16]		2017 (n = 145) [17]		2019 (n = 194) [5]		2020 (n = 595) [18]		Present (n = 410)	
	n	%	n	%	n	%	n	%	n	%	n	%
IGHV4-39	2	3.1%	3	6.5%	5	3.4%	11	5.7%	39	6.6%	21	5.1%
IGHV3-74	3	4.6%	0	0%	<3	<2.0%	14	7.2%	<19	<3.0%	13	3.2%
IGHV1-69	1	1.5%	1	2.2%	<3	<2.0%	11	5.7%	37	6.2%	28	6.8%
IGHV3-21	2	3.1%	2	4.3%	4	2.8%	<11	<5.0%	19	3.2%	12	2.9%
IGHV3-30	4	6.2%	1	2.2%	<3	<2.0%	12	6.2%	34	5.7%	15	3.7%
IGHV3-07	3	4.6%	1	2.2%	20	13.8%	18	9.3%	45	7.6%	26	6.3%
IGHV4-34	8	12.3%	10	21.7%	28	19.3%	11	5.7%	59	9.9%	54	13.2%
IGHV4-59	7	10.8%	1	2.2%	5	3.4%	13	6.7%	26	4.4%	14	3.4%
IGHV3-23	5	7.7%	4	8.7%	20	13.8%	20	10.3%	61	10.3%	36	8.8%
IGHV4-61	5	7.7%	1	2.2%	<3	<2.0%	<11	<5.0%	<19	<3.0%	11	2.7%
IGHV3-11	5	7.7%	1	2.2%	<3	<2.0%	<11	<5.0%	<19	<3.0%	11	2.7%
IGHV3-48	1	1.5%	4	8.7%	<3	<2.0%	<11	<5.0%	23	3.9%	16	3.9%

**Table 3:** Cytogenetic aberrations and immunophenotypes distribution among the three molecular subgroups.

	MG-1	MG-2	MG-3	*p* Value
Age, median	63.7%	64.1%	65.3%	0.20
Sex, male/female ratio	1.7	3.5	1.9	0.11
AK (pos, %)	6.9%	8.1%	23.1%	<0.001
CK (pos, %)	0.8%	5.4%	5.8%	0.06
Trisomy 12 (pos, %)	11.9%	11.1%	26.3%	0.053
Del(11q22.3) (pos, %)	5.5%	0.0%	14.6%	0.07
Del(17p13.1) (pos, %)	0.0%	0.0%	12.6%	<0.001
Del(13q14.2) (pos, %)	30.6%	25.0%	17.8%	0.26
CD5 (pos, %)	100.0%	98.1%	99.8%	0.06
CD10 (neg, %)	100.0%	100.0%	99.8%	0.78
CD23 (pos, %)	99.5%	94.3%	98.5%	0.03
FMC7 (neg, %)	93.0%	66.0%	93.5%	<0.001
CD22 (neg/dim, %)	42.5%	35.5%	40.1%	0.79
CD79b (neg/dim, %)	77.4%	53.3%	70.7%	0.007
sIg (neg/dim, %)	36.9%	29.6%	32.3%	0.44
CD200 (pos, %)	97.5%	73.2%	96.4%	<0.001

## Data Availability

The datasets analysed in the current study are available from the corresponding author on reasonable request.

## Supplementary Materials

The following supporting information can be downloaded at: https://www.mdpi.com/article/10.3390/jcm12072667/s1, Table S1: Gene list of 175 genes associated with hematological malignancy (175-Panel); Table S2: Testing results of 761 CLL/SLL cases; Table S3: Testing results of 74 MCL cases.
